# The Use of Manual Vacuum Aspiration in the Treatment of Incomplete Abortions: A Descriptive Study from Three Public Hospitals in Malawi

**DOI:** 10.3390/ijerph15020370

**Published:** 2018-02-21

**Authors:** Maria Lisa Odland, Gladys Membe-Gadama, Ursula Kafulafula, Geir W. Jacobsen, James Kumwenda, Elisabeth Darj

**Affiliations:** 1 Department of Public Health and Nursing, Norwegian University of Science and Technology, NO-7491 Trondheim, Norway; geirjacobsen@ntnu.no (G.W.J.); elisabeth.darj@ntnu.no (E.D); 2 Queen Elizabeth Central Hospital, Blantyre, Malawi; glachime@yahoo.com; 3 Kamuzu College of Nursing, Blantyre, Malawi; ursulakafulafula@kcn.unima.mw; 4 Mangochi District Hospital, Mangochi, Malawi; kumwendajk@gmail.com; 5 Department of Woman´s Health and Children´s Health, Uppsala University, SE-751 85 Uppsala, Sweden; 6 Department of Gynecology, St. Olav´s Hospital, NO-7030 Trondheim, Norway

**Keywords:** incomplete abortions, unsafe abortions, uterine evacuation, post-abortion care, manual vacuum aspiration, female health, maternal mortality, low-income countries, Malawi

## Abstract

Malawi has a high maternal mortality rate, of which unsafe abortion is a major cause. About 140,000 induced abortions are estimated every year, despite there being a restrictive abortion law in place. This leads to complications, such as incomplete abortions, which need to be treated to avoid further harm. Although manual vacuum aspiration (MVA) is a safe and cheap method of evacuating the uterus, the most commonly used method in Malawi is curettage. Medical treatment is used sparingly in the country, and the Ministry of Health has been trying to increase the use of MVA. The aim of this study was to investigate the treatment of incomplete abortions in three public hospitals in Southern Malawi during a three-year period. All medical files from the female/gynecological wards from 2013 to 2015 were reviewed. In total, information on obstetric history, demographics, and treatment were collected from 7270 women who had been treated for incomplete abortions. The overall use of MVA at the three hospitals during the study period was 11.4% (95% CI, 10.7–12.1). However, there was a major increase in MVA application at one District Hospital. Why there was only one successful hospital in this study is unclear, but may be due to more training and dedicated leadership at this particular hospital. Either way, the use of MVA in the treatment of incomplete abortions continues to be low in Malawi, despite recommendations from the World Health Organization (WHO) and the Malawi Ministry of Health.

## 1. Introduction

Malawi, in South East Africa, is one of the poorest countries in the world, with a population of 18 million inhabitants, limited resources and an impoverished health care system [1,2]. Consequently, it has one of the highest maternal mortality rates in the world, with 439 maternal deaths per 100,000 live births [3]. Unsafe abortion is a prominent cause of this [4] and accounts for 6–30% of all maternal mortalities in Malawi [5,6,7,8]. Even though there are ongoing discussions to liberalize the law, abortion is only legal when it is necessary to save a pregnant woman’s life [9,10]. Unsafe abortions make up the majority of abortions in Malawi, with the most recent estimate being 140,000 induced abortions annually [11,12]. Products of conception retained inside the uterus is one of the most common complications after an abortion, and almost 13% of the women receiving post-abortion care in Malawi in 2009 presented with incomplete abortions [13]. Additionally, incomplete abortions can lead to more serious complications such as haemorrhage, sepsis, and in the worst-case scenario, death [14,15]. Incomplete abortions are generally treated with surgical or medical uterine evacuation [16]. In Malawi, medical evacuation is not frequently used [17]. Surgical evacuation can be carried out by using vacuum aspiration or dilatation and curettage (D&C) [16]. Curettage usually requires general anaesthesia, an operating theatre, and the efforts of a medical doctor or clinical officer [16,18]. Comparatively, manual vacuum aspiration (MVA) is a safe and cheap method that can be performed by authorized nurses without the use of general anaesthesia or access to electricity [16,18]. The World Health Organization (WHO) guidelines and a Cochrane Library review concluded that vacuum aspiration is the preferred surgical method for uterine evacuation after an incomplete abortion in the first trimester [16,18,19]. Manual vacuum aspiration is faster, less painful, and is associated with less blood loss and fewer complications than D&C [16]. A recent Malawian study showed that the median cost per D&C intervention was 29% higher than the cost per MVA ($63 versus $49 United States Dollars) [20]. This is important in a health care system with limited resources. Nevertheless, in 2014, a study showed that the use of MVA declined during the 2008 to 2012 time period, while the use of D&C increased in selected parts of the country [17]. In 2012, MVA use accounted for just under 5% of uterine evacuations, with D&C use accounting for about 95%, and medical treatment for <1% of uterine evacuations [17]. Even though MVA equipment is on the government standard equipment list, and its use is officially promoted, many health facilities do not use it to evacuate incomplete abortions [21]. A qualitative study in two hospitals with a declining use of MVA, suggested that several factors influenced post-abortion care and MVA use; lack of training, and shortage of equipment and human resources were mentioned as major limiting factors [22]. 

Encouraging the use of MVA could help reduce obstetric complications and optimize resources. Considering the quality of existing data, the decline in the use of MVA in the southern part of Malawi and the reasons for this decline, new and updated information is required before a larger educational intervention can be initiated [17]. Accordingly, the aim of this study was to investigate how women with incomplete abortions were treated in three public hospitals in Southern Malawi between 2013 and 2015.

## 2. Materials and Methods

### 2.1. Study Design and Setting

The study was designed to identify which methods were used to manage incomplete abortions for women seeking post-abortion care in public hospitals in the southern part of Malawi; this constitutes a follow-up to a previous survey [17]. We chose a retrospective descriptive design that involved reviewing hospital files for a chosen time-period.

The study was conducted at two district hospitals, Chiradzulu and Mangochi, and the Queen Elizabeth Central Hospital (QECH). While the majority of post-abortion care cases are treated in public hospitals [13], the QECH is the referral hospital for the whole southern region of Malawi. Hence, a large number of women with incomplete abortions are treated at the QECH.

### 2.2. Study Population

All records from patients admitted in the three study hospitals were routinely stored after discharge and could be accessed by the clerk in charge at each hospital. All files from the female/gynecological ward, between 1 January 2013 and 31 December 2015, at the three hospitals were retrieved and reviewed. Women who had been treated for incomplete abortions were included.

### 2.3. Inclusion and Exclusion Criteria

Fetal death up to 28 weeks of gestation is classified as a miscarriage in Malawi, and therefore all pregnant women in this category were included. Women admitted for all other reasons, as well as women who were not offered any post-abortion treatment at all, were excluded. Since complications after a spontaneous miscarriage and an induced abortion are hard to distinguish, and are mostly unreported, these cases were not separated. Manual vacuum aspiration should preferably only be used in the first trimester, and may potentially be used up to 14-weeks of gestation [18]. However, mothers of higher gestational ages were included, as many of these women may have had residual amounts of retained products that might have been treated with MVA if they had been examined properly prior to surgery.

### 2.4. Data Collection

Data were taken from the female/gynecological ward records by a team of three research assistants, including nurses and midwives familiar with medical terms. The process was managed by a medical doctor, who also served as the principal investigator. The same data extraction tools were employed at all three study sites. Demographic data (residence, age, marital status, level of education, religion, and occupation), reproductive history (gravity, parity, number of children still alive, and gestational age), length of hospital stay, and type of evacuation were retrieved for each patient.

### 2.5. Study Period

Data collection was conducted during the period from 1 April 2016 to 31 May 2016.

### 2.6. Statistical Analysis

Data were analyzed using IBM SPSS Statistics version 22 (Armonk, New York, USA). Values are given as proportions (percent) with their 95% confidence interval (CI), and age is reported as the mean and standard deviation (SD).

### 2.7. Ethics Statement

Ethical approval was granted by the local Malawian College of Medicine Research and Ethics Committee (COMREC) P.06/15/1748, and the Regional Committee for Medical and Health Research Ethics Central Norway (REC Central), 2015/455/REK. Permission to access individual patient records was granted by the District Health Officers at Mangochi and Chiradzulu District Hospitals, and the Head of the Department of the Obstetric and Gynecological ward at QECH. All patient information was anonymized and de-identified prior to analysis.

## 3. Results

Demographic characteristics of the three annual study cohorts, and for all years, are provided in Table 1. The overall mean maternal age was 24.8 (SD 6.5) years, and the average number of offspring was 1.4 (SD 1.6), with 5.6% having more than five offspring (range 1–12). About one-third of the mothers (29.5%, 95% CI, 28.4–30.7) had no previous births, 85.6% (95% CI, 83.6–87.4) were married, 4.3% (95% CI, 2.9–6.2) had a higher education, and about one-third (33.9%; 95% CI, 30.9–36.9) had gainful employment. Islam was the most common religion (30%), followed by Christian groups including Roman Catholics (18%), and members of the Church of Central Africa Pentecost (12%). There were no obvious differences in demographic characteristics over the years (Table 1).

The overall use of MVA in the three study hospitals (*n* = 7270) from 2013 to 2015 was 11.4% (Table 2). Correspondingly, surgical evacuation with D&C was used in 86.4% of the cases. Medical treatment with Misoprostol was used in 1.4 % (95% CI, 1.1–1.7) of the cases. A combination of suction and curettage was only used in cases with molar pregnancies at QECH, which included 0.5% (95% CI, 0.4–0.7) of the cases. In addition, a few cases (0.3% (95% CI, 0.2–0.4)) had laparotomy and hysterectomy. Only MVA and D&C are included in Table 2 and Table 3.

Overall, there was an increase in the use of MVA at all three hospitals, from 6.9% (95% CI, 5.9–8.0) in 2013, to 17.4% (95% CI, 15.9–19.0) in 2015 (as seen in Table 2). This increase occurred mostly in the Mangochi hospital (9.1% in 2013 to 40.6%); in the other two hospitals there was a marginal increase in the use of MVA, from 8.4% to 14.4% at Chiradzulu, and 5.9% to 11.8% at QECH. 

**Figure 1:**
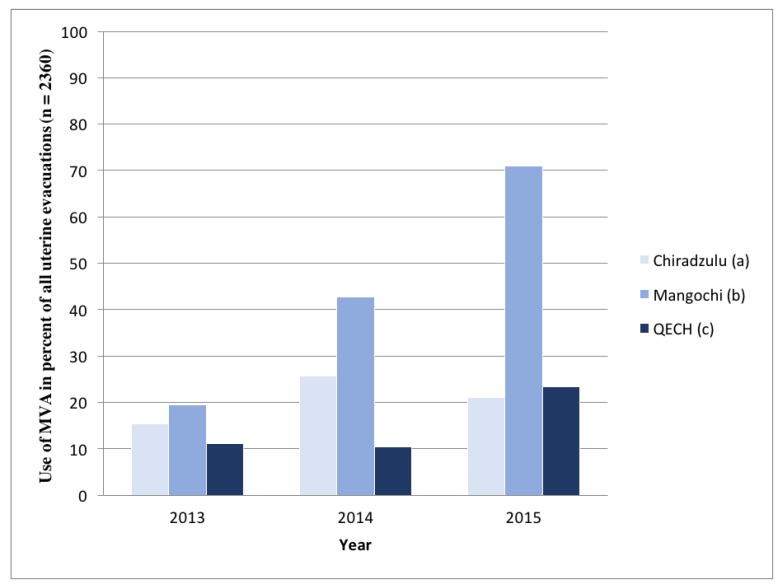
Use of manual vacuum aspiration (MVA) for removal of retained products of conception in first-trimester incomplete abortions, by year and specific Malawi hospital (2013 to 2015). (**a**) Chiradzulu District Hospital; (**b**) Mangochi District Hospital; (**c**) Queen Elizabeth Central Hospital.

Table 3 and Figure 1 show first-trimester abortions only. Of all the incomplete abortions in the first-trimester, 21.4% (95% CI, 19.7-23.0) were treated with MVA (Table 3). However, in 2015 the Mangochi District Hospital used MVA to treat 70.9% of the patients with first-trimester abortions (Table 3 and Figure 1).

During the last six months of 2015, the Mangochi District Hospital treated between 80% and 90% of all first-trimester abortions with MVA (not shown in Table 3). Furthermore, the Mangochi District Hospital experienced a 10.4% decline in hospital-based maternal mortality from 2013/2014 to 2015. In addition, the Mangochi District Hospital had no maternal deaths due to abortion in 2015. At the two other hospitals, there was no decline in hospital-based maternal mortality, and at QECH there was a slight increase in maternal deaths due to abortion.

## 4. Discussion

The overall use of MVA as a part of post-abortion care in the southern districts of Malawi during the 2013 to 2015 period was 11.4%, which is lower than the overall use of MVA from 2008 to 2012 [17]. Nevertheless, we found an increase in the use of MVA in all three hospitals in 2015 compared to 2013 [17]. One hospital, namely the Mangochi District Hospital, was primarily responsible for the overall study outcome, and showed more than a 50% increase in the use of MVA in the treatment of first trimester abortions.

We could not distinguish between miscarriages and induced abortions, and therefore these were not studied separately. However, there were abortion cases that showed signs of having been induced with foreign objects, and a few women admitted that they had taken drugs or local herbs to induce termination of their pregnancy. Also, several patients presented with symptoms of miscarriage in the second trimester. Considering that most miscarriages occur in the first trimester [23], and that two-thirds of the women in our study presented with symptoms of a second-trimester miscarriage, it seems reasonable to assume that most of the latter abortions were induced. Nevertheless, most women (*n* = 2036) were in their 13^th^ week of gestation when they presented with symptoms. 

A limitation of the present study is its retrospective nature, with the use of existing hospital files as the data source. Since all records were kept on paper, there is the possibility that some information may have been lost. In our previous study, for the 2008 to 2012 period, it was only possible to locate 5000 women that had been registered for treatment of incomplete abortions at the three hospitals [17]. In the current study, more than 7000 files were retrieved over the three-year period, which may indicate that the hospital records had been retained more effectively and accurately over the last few years. The fact that all files were sorted by month supports this. Moreover, all three hospitals had a designated clerk in charge of the records, which facilitated our task. Possibly, an increase in interest may have improved record keeping among health care personnel. Even so, we acknowledge that some files may have been lost, as there is some missing data.

A strength of retrospective data collection is the reduction of selection bias; the care providers did not know that their information would be used in research. Hence, they chose their treatment without being influenced by the researchers or any pre-study hypothesis. Furthermore, a selected team of health personnel was supervised by one principal investigator so that the same search protocol was employed throughout the data collection period. In part, our study was carried out by the same local teams as in 2012, and we believe this limited any errors due to misclassification [17].

Because our project took place in only three hospitals in Southern Malawi, our results may not be generalizable to the whole country, even though they were comparable to those in our previous study [17]. Together, these research efforts allowed for the observation of trends in the treatment of incomplete abortions over a longer period. 

There was an increase in the use of MVA in all the hospitals, although the hospital of Mangochi was the only one that had a documented major improvement. In past years, there has been a general shift in policy by the Malawi Ministry of Health to encourage a reduction in maternal deaths, and MVA has been a promoted treatment, as it leads to fewer complications. The Mangochi District Hospital encouraged staff to attend MVA training sessions organized by the Ministry of Health; the staff in turn trained their colleagues. The hospital management also promoted and encouraged the use of MVA. The Mangochi District Hospital is the only hospital that adopted the guidelines fully, and treated almost 90% of first-trimester abortions with MVA by the end of 2015. The results are promising, and we speculate that this accounts for the decline in maternal mortality [16,19]. In fact, there were no maternal deaths due to abortion at this hospital in 2015. The other study hospitals did not experience a decrease in maternal mortality and had more deaths due to abortion in 2015. Even so, change in maternal mortality is not necessarily directly linked to the use of MVA, but more use of MVA could have reflected improvements in other parts of the hospital, such as improved obstetric and postnatal care.

Medical treatment with Misoprostol is increasingly being used to treat incomplete abortions and is a cheap and safe way to induce contractions of the uterus and an evacuation in a natural way [20,24,25]. However, the QECH was the only hospital that used medical treatment, in just 3.2% (95 CI, 2.6–4.5) of the cases in 2015; medical treatment was mostly used to prepare patients for surgical treatment. Low use of medical evacuation of the uterus could be due to limited resources and a fear of misuse in inducing abortions. However, medical evacuation of the uterus is more often incomplete [26,27,28], and patients are supposed to return for a check-up to be rescanned and ensure that the uterus is empty [27,28]. In Malawi, patients may not come back to be rescanned due to long waiting times, and lack of transportation and money, which could then lead to incomplete septic miscarriages associated with high morbidity and mortality. Hence, health personnel are more comfortable evacuating the uterus surgically to ensure that the patient will not be lost to follow-up. If surgical treatment is maintained as the best option, which seems to be the case in Malawi at the moment, then MVA is the safest and cheapest way to treat incomplete abortions in the first trimester [16,20].

Maternal mortality is still high in Sub-Saharan Africa and unsafe abortion is a major cause that in turn is closely related to the abortion laws [4,6,7,8]. Legalizing abortion has led to a reduction in maternal mortality in several countries, such as Romania and South Africa [29]. Fortunately, Malawi is considering moderating their restrictive abortion laws. Even so, an immediate change making abortion accessible to women is not likely to happen in the near future. In the meantime, other measures need to be taken to reduce maternal mortality. Using MVA rather than D&C can make the treatment of incomplete abortions safer and cheaper, which is important in a health care system that is already frail due to limited resources and staff.

## 5. Conclusions

While one of the study hospitals increased their use of MVA significantly, the other two hospitals continued to use D&C as a primary treatment for incomplete abortions. The observed trend of increased use of MVA could be due to training and better access to equipment, as seen in other developing countries [30,31,32], and should be investigated further in Malawi.

## Figures and Tables

**Table 1:** Available information on demographic characteristics of women treated for incomplete abortions in the three hospitals in Malawi during the period of 2013 to 2015 (*n* = 7270) ^1^.

Characteristics	2013 (*n* = 2307)	2014 (*n* = 2561)	2015 (*n* = 2402)	All Years (*n* = 7270)
Mean age (SD) years	24.9 (6.4)	24.8 (6.6)	24.9 (6.5)	24.8 (6.5)
**Pregnancy history**				
Primigravida	479 (26.6)	655 (31.9)	561 (29.8)	1695 (29.5)
Multigravida	1321 (73.4)	1397 (68.1)	1324 (70.2)	4042 (70.5)
**Number of living children**				
None	390 (36.4)	609 (40.7)	446 (39.9)	1445 (39.2)
1	238 (22.2)	303 (20.3)	232 (20.8)	773 (21.0)
2+	442 (41.4)	584 (39.0)	440 (39.3)	1466 (39.8)
**Gestational length**				
1st trimester	737 (34.8)	851 (36.1)	772 (35.1)	2360 (35.3)
>1st trimester	1382 (65.2)	1507 (63.9)	1429 (64.9)	4318 (64.7)
**Marital status**				
Unmarried ^2^	38 (12.4)	68 (13.7)	88 (16.1)	194 (14.4)
Married	268 (87.6)	427 (86.3)	458 (83.9)	1153 (85.6)
**Hospital Admission** ^3^				
Rural facility	825 (35.8)	909 (35.5)	867 (36.1)	2601 (35.8)
Urban facility	1482 (64.2)	1652 (64.5)	1535 (63.9)	4669 (64.2)
**Educational level**				
None	1 (1.3)	6 (2.9)	9 (2.5)	16 (2.5)
Primary	42 (53.8)	107 (51.7)	181 (50.1)	330 (51.1)
Secondary	34 (43.6)	86 (41.5)	152 (42.1)	272 (42.1)
Tertiary	1 (1.3)	8 (3.9)	19 (5.3)	28 (4.3)
**Occupation**				
None	74 (34.7)	158 (44.9)	159 (40.0)	391 (40.6)
Housewife	48 (22.5)	41 (11.6)	37 (9.3)	126 (13.1)
Student	634 (16.0)	37 (10.5)	49 (12.3)	120 (12.4)
Gainful employment ^4^	57 (26.8)	116 (33.0)	153 (38.4)	237 (33.9)

^1^ Numbers are given as *n* (%) unless otherwise indicated. ^2^ The unmarried group includes: being single, separated, divorced, or widowed. ^3^ Rural: Chiradzulu and Mangochi hospitals. Urban: Queen Elizabeth Central Hospital (QECH). ^4^ Gainful employment: cleaner, farmer, businesswoman, policewoman, and other.

**Table 2:** Surgical methods used for removal of retained products of conception after incomplete abortions ^1^, by year and hospital in the three Malawi hospitals during 2013 to 2015 ^2,3^.

Year	Type of PAC	Chiradzulu (*n* = 1117)	Mangochi (*n* = 1484)	QECH ^4^ (*n* = 4669)	All Hospitals (*n* = 7270)
2013 (*n* = 2307)	MVA ^5^	8.4 (5.5–12.0)	9.1 (6.8–12.0)	5.9 (4.7–7.2)	6.9 (5.9–8.0)
	D&C ^6^	91.3 (87.6–94.2)	89.5 (86.5–92.0)	91.5 (90.0–92.9)	91.0 (89.8–92.2)
2014 (*n* = 2561)	MVA ^5^	14.9 (11.5–19.0)	20.5 (17.2–24.2)	5.2 (4.2–6.4)	9.8 (8.7–11.1)
	D&C ^6^	85.1 (81.0–88.5)	78.9 (75.2–82.3)	92.4 (91.0–93.6)	88.5 (87.2–89.7)
2015 (*n* = 2402)	MVA ^5^	14.4 (11.2–18.0)	40.6 (35.9–45.4)	11.8 (10.2–13.5)	17.4 (15.9–19.0)
	D&C ^6^	85.6 (82.0-88.8)	59.2 (54.4–63.9)	83.8 (81.9–85-7)	79.8 (78.1–81.4)
All years (*n* = 7270)	MVA ^5^	12.9 (11.0-15.0)	22.4 (20.3-24.6)	7.6 (6.8–8.4)	11.4 (10.7–12.2)
	D&C ^6^	87.0 (84.9-88.9)	76.9 (74.7-79.0)	89.3 (88.4–90.2)	86.4 (85.6–87.2)

^1^ Gestation up to 28 weeks. ^2^ Numbers are given as percentages (95% CI), unless otherwise indicated. ^3^ Suction and curettage, laparotomy, and medical treatment were not included. ^4^ Queen Elizabeth Central Hospital (QECH). ^5^ Manual vacuum aspiration (MVA). ^6^ Dilatation and Curettage (D&C).

**Table 3:** Surgical methods used for removal of retained products of conception for first-trimester incomplete abortions ^1^ in three Malawi hospitals by year for the 2013 to 2015 period ^2,3^.

Year	Type of PAC	Chiradzulu (*n* = 372)	Mangochi (*n* = 475)	QECH ^4^ (*n* = 1513)	All Hospitals (*n* = 2360)
2013 (*n* = 737)	MVA ^5^	15.3 (9.5–22.9)	19.4 (13.8–25.1)	11.2 (8.4–14.5)	13.8 (11.4–16.5)
	D&C ^6^	84.7 (77.1–90.5)	80.0 (73.3–85.7)	86.3 (82.7–89.4)	84.5 (81.7–87.1)
2014 (*n* = 851)	MVA ^5^	25.6 (18.2–34.2)	42.8 (35.1–50.7)	10.4 (8.0–13.2)	18.9 (16.3–21.7)
	D&C ^6^	74.4 (65.8–81.8)	57.2 (49.3–64.9)	87.9 (84.9–90.4)	79.9 (77.1–82.5)
2015 (*n* = 772)	MVA ^5^	21.1 (14.3–29.4)	70.9 (62.4–78.4)	23.3 (19.7–27.2)	31.2 (28.0–34.6)
	D&C ^6^	78.9 (70.6–85.7)	29.1 (21.6–37.6)	70.7 (66.5–74.6)	64.8 (61.3–68.1)
All years (*n* = 2360)	MVA ^5^	20.7 (16.7–25.2)	42.1 (37.6–46.7)	15.0 (13.2–16.9)	21.4 (19.7–23.1)
	D&C ^6^	79.3 (74.8–83.3)	57.7 (53.1–62.2)	81.6 (79.5–83.5)	76.4 (74.6–78.1)

^1^ Abortions in first trimester included. ^2^ Numbers are given as percentages (95% CI) unless otherwise indicated. ^3^ Suction and curettage, laparotomy and medical treatment were not included; ^4^ Queen Elizabeth Central Hospital (QECH). ^5^ Manual vacuum aspiration (MVA). ^6^ Dilatation and Curettage (D&C).
